# ^15^N Natural Abundance of C3 and C4 Herbaceous Plants and Its Response to Climatic Factors along an Agro-Pastoral Zone of Northern China

**DOI:** 10.3390/plants11243526

**Published:** 2022-12-14

**Authors:** Xianzhao Liu, Yang Li, Yong Zhang, Qing Su, Teng Feng, Yan Song

**Affiliations:** 1 School of Earth Science and Spatial Information Engineering, Hunan University of Science and Technology, Xiangtan 411201, China; 2 College of Life Science, Hunan University of Science and Technology, Xiangtan 411201, China

**Keywords:** agro-pasture zone in North China (APZNC), climatic factors, C3/C4 herbs, nitrogen stable isotope

## Abstract

The nitrogen isotope composition of plants (δ^15^N) can comprehensively reflect information on climate change and ecosystems’ nitrogen cycle. By collecting common herbs and soil samples along the 400 mm isoline of mean annual precipitation (MAP) in the agro-pastoral zone of North China (APZNC) and measuring their δ^15^N values, the statistical characteristics of foliar δ^15^N of herbs and the responses of foliar δ^15^N to the MAP and mean annual temperature (MAT) were analyzed. The results showed that: (1) the δ^15^N values of all herbs investigated varied from −5.5% to 15.25%. Among them, the δ^15^N value range of C3 herbs (−5.5~15.00%) was wider than that of C4 herbs (−2.17~15.25%), but the average value (3.27%) of C3 herbs was significantly lower than that of C4 herbaceous plants (5.55%). This difference provides an important method for identifying plants of different photosynthetic types by nitrogen isotope technology. (2) Along the transect from northeast to southwest, the δ^15^N of both C3 and C4 herbs decreased with the increase in the MAP, but not significantly for C3 herbs. The inverse relationship between the nitrogen isotopic signatures of herbs and MAP is consistent with previous studies. However, the MAP in the APZNC is found to only explain a small amount of the observed variance in the δ^15^N herbs (C3 herbs: 10.40%; C4 herbs: 25.03%). (3) A strong negative relationship was found between δ^15^N of herbs and MAT across the transect (C3 herbs: −0.368%/°C; C4 herbs: −0.381%/°C), which was contrary to the global pattern and some regional patterns. There was no significant difference in the δ^15^N responses of two different photosynthetic herbs to temperature, but the effect of temperature on the variances of δ^15^N of C3 and C4 herbs was significantly greater than that of precipitation. This suggests that temperature is a key factor affecting foliar δ^15^N of herbs in this transect. The above findings may be of value to global change researchers studying the processes of the nitrogen cycle and gaining an insight into climate dynamics of the past.

## 1. Introduction

Nitrogen (N) has long been considered as one of the most extensive nutrients that constrain plant growth, maintenance, and reproduction in many terrestrial ecosystems, and its cycle affects almost all aspects of ecosystem functions [1,2,3]. Since the natural abundance of ^15^N in plant tissue is a combined result of a series of biogeochemical processes and environmental changes, the nitrogen isotope composition (δ^15^N) in plants can record a series of climate and environmental information related to physio-ecological processes of plants to a certain extent, and become an focal tool to infer past short-term (e.g., annual time scales) variations in the ecological environment or to indirectly indicate ecosystem function and N-cycling processes that are difficult to measure directly [2,4,5,6,7,8,9]. These applications are based mainly on the general response pattern of plant δ^15^N to ecological and climatic (precipitation and temperature) gradients at local to global scales. At present, regional and global patterns of N isotopes in different terrestrial plants have been extensively reported along a geographic climatic gradient [10,11,12,13]. The general view is that on a regional and global scale, plant δ^15^N increased with increasing mean annual temperature (MAT) and decreasing mean annual precipitation (MAP) [14,15,16,17]. This pattern was explained by the fact that warm, dry sites have a larger proportion of nitrogen losses through fractionating and more open N cycling, whereas cold and wet sites seem to be more effective in preserving and recycling mineral N. Subsequently, a global study of more than 11,000 non-N2-fixing plants demonstrated that foliar δ^15^N increased with decreasing MAP and increasing MAT, but only for those sites with MAT > −0.5 °C [10]. Owing to the positive correlation obtained at the local scale between N availability and plant δ^15^N, the global relation of climate and plant δ^15^N was interpreted as higher N availability in warm, dry sites. Contrary to the above-mentioned study, Pardo et al. found a positive relationship between foliar δ^15^N and rainfall in the range of 500–1800 mm across northeastern North America, Colorado, Alaska, Southern Chile and Europe [18]. Feng et al. and Sah et al. also concluded that the δ^15^N values in plant and soil increased with increasing MAP and decreasing MAT on a regional scale [19,20]. Moreover, the results from the Loess Plateau of China showed that foliar δ^15^N decreased with increasing MAT and MAP [21], while over eastern China, the foliar δ^15^N of oriental oak increased significantly with increasing MAP and MAT [22]. These confusing results above might be attributed to the differences in plants’ functional group level (e.g., growth forms, life history and C3 and C4 photosynthetic pathways), environmental control variables and geospatial scales. Over the past few decades, a large number of advances have been made in quantifying foliar δ^15^N patterns on a local and global scale as well as in the mechanisms that underlie these patterns, but our mechanistic understanding of the patterns of natural ^15^N abundance in plants and their underlying causes is still developing. In particular, since most previous studies on δ^15^N variation in plants were focused on woody plants, we still do not know the response patterns of δ^15^N in herbaceous plants with C3 and C4 photosynthetic pathways to some geographical and climatic variables, thus hindering the correct interpretation of N cycling, structure and function changes in grassland ecosystems. Although some scholars have also studied the nitrogen isotopes of C3 and C4 plants along climatic and land-use gradients in many regions (e.g., Southern Africa, South China and Tengger Desert of China), in their study, most C3 plants used were trees and shrubs, except for C4 plants, which were all herbs [4,23,24,25]. For example, Ruiz-Navarro et al. measured foliar δ^15^N in three plant species representing C3 and C4 plant functional types in a topographically complex semi-arid ecosystem, of which the C3 plants investigated were trees and shrubs, and only the C4 plants were grass [23]. Another example was that Zhao et al. examined the δ^15^N values of the coexistent C3 (shrubs) and C4 (herbs) species occurring in different aged artificial sand-binding microhabitats, as well as in natural habitats at the southeastern margin of the Tengger Desert, China [24]. Since plant species with different growth forms (herbs, shrubs, trees and lianas) differ widely in key functional traits (including rooting depth, mycorrhizal association type and photosynthetic nitrogen utilization strategy), the differences in preferred N uptake forms or internal N metabolism may lead to great differences in plant δ^15^N responses to environmental factors, which might give rise to significant differences in foliar δ^15^N values between C3 and C4 plants [22,24,25,26,27]. Some studies have shown that there were significant differences between C3 and C4 plants in δ^15^N values. For example, higher δ^15^N values in C4 plants (compared to their coexisting C3 species) were found in the Mediterranean region and Western Australia, but C3 plants had higher δ^15^N values than C4 plants in Southern Africa and the Tengger Desert of China [4,24,25,28]. The above-mentioned inconsistent results may be caused by the failure to distinguish trees, shrubs and herbs when comparing the δ^15^N values of plants with different photosynthetic pathways. This implies that previous studies mostly focused on the plant growth form level and did not really reveal the patterns of variations in foliar δ^15^N of C3 and C4 herbs. Consequently, it is needed to explore the δ^15^N responses for C3 and C4 herbs along an environmental gradient and to seek relevant affecting factors. This can further promote our understanding of how foliar δ^15^N in herb species responds to environmental factors and help us explain the changes in functions and regulatory mechanisms of key factors involved in the N cycling of grassland ecosystems. Contrast to previous studies, our research has made important contributions in three aspects. First, by comparing the differences in δ^15^N values between C3 and C4 herbs, a new method is provided for identifying plants with different photosynthetic types by nitrogen isotope technology. Second, based on the response of nitrogen isotopes of C3 and C4 herbs to temperature and precipitation changes (if this pattern is indeed robust), it can provide basic data for using stable isotopes to explain the process of the nitrogen cycle in temperate grasslands of North China. Third, because the δ^15^N of C3 and C4 herbs contains a lot of climate–environmental information, this will provide a new idea for paleoclimatologists to use plant δ^15^N as a climate proxy to deeply explore the past climate dynamics and paleoenvironment reconstruction.

The agro–pastoral zone of Northern China (FPZNC) is a relatively independent geographical region. The complex and diverse topography of the study area consists of gently rolling hills, grasslands, sandy land, and platforms, with an altitude of 650 m to 1800 m. Its natural environment variables such as climate, vegetation and soil have distinct transitional characteristics. The sensitivity of the geochemical cycle within the ecosystem to temperature and humidity fluctuations makes this region an obvious indicator of environmental changes. This region belongs to a typical temperate continental semi-arid monsoon climate: dry and hot in summer, cold in winter, sunny in autumn, strong winds in spring and frequent sandstorms. The mean annual precipitation (MAP) ranges between 345 mm and 443 mm and the mean annual temperature (MAT) varies from −6.1 °C to 8.9 °C, with a decreasing trend from northeast to southwest [29]. In this area, more than 60% of the annual rainfall occurs in the summer season from June to August owing to its climate condition and geographical location. The natural vegetation is consistent with semi-humid, semi-arid and semi-desert climates extending from the northeast to the southwest, and is expressed as continuous changes in vegetation from meadow steppes to typical steppes and desert steppes from east to west. The C3 and C4 herbs with different photosynthetic pathways coexist in the FPENC, of which C3 herbs are dominant and widely distributed, and C4 herbs are limited in number [29]. The soil types formed under unique climatic conditions are mainly chestnut soil, loess soil and chernozem. The unique features of this region, including a continuum of mesic to xeric grassland types, distinct climatic gradients and relatively light human disturbance, provide ideal conditions to explore the response patterns of the natural abundance of ^15^N in C3 and C4 herbs along a regional environmental gradient. Although some scholars have explored the relationship between plant δ^15^N and environmental factors in the APZNC, the scope of the research is limited to a typical section [30,31]. In such an agro-pastoral ecotone that is thousands of kilometers long, there are few reports on the systematic investigation of the relationships between δ^15^N of C3 and C4 herbs and environmental variables. This study systematically investigates the variations in δ^15^N of C3 and C4 herbs along a precipitation and temperature gradient in the APZNC, and addresses two scientific questions: (1) whether are there significant differences between the δ^15^N of C3 and C4 herbs from the same sites in the APZNC due to differences between photosynthetic pathways? (2) How do the δ^15^N values of C3 and C4 herbs in this area respond to driving factors such as changes in temperature and precipitation, and is there any difference in the response patterns?

## 2. Results and Discussion

### 2.1. Comparison of Foliar δ^15^N between C3 and C4 Herbs

**Figure 1:**
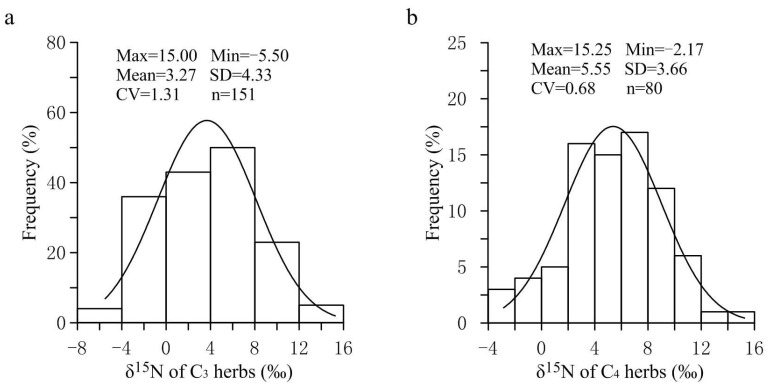
Frequency distribution of δ^15^N for C3 and C4 herbs in the study area. (**a**) Nitrogen isotopic frequency of C3 Herbs. (**b**) Nitrogen isotopic frequency of C4 Herbs.

As shown in Figure 1, the frequency distribution of foliar δ^15^N values of C3 and C4 herbs in the APZNC was of unimodal type. The foliar δ^15^N values of overall herbs ranged from −5.50% to 15.25%, with a coefficient of variation (CV) of 1.05, indicating that the δ^15^N values of herbs had a large spatial variability in the study area. Among them, the C3 herbs showed a wider range of δ^15^N values (−5.50–15.00%) than C4 herbs (−2.17–15.25%), and the CV of the former was 1.9 times that of the latter, which might be related to the fact that the number of C3 herb species collected in this study was far more than that of C4 herb species (Table 3). The reason is that in the APZNC, although C3 and C4 herbs are widely distributed, the number of C4 herbs is extremely limited. Further analysis found that in our nitrogen isotope data set, more than 95.0% of the foliar δ^15^N values fell in the range of −4.0% to 12.0%, which is basically consistent with the previously reported range of plant δ^15^N values in North China (C3 plants: −5.1–13.0%; C4 plants: −3.2–12.4%) [11]. However, compared with the range of foliar δ^15^N for 11,000 plants worldwide from −10.0% to 17.0% [10], the range of foliar δ^15^N investigated in the APZNC is much more concentrated. There may be two reasons for this phenomenon. First, the samples in this study were all from the transition zone between the semi-arid region and the semi-humid region, so the climate conditions in our study area were relatively simple compared with those in the rest of the world. Second, the plant samples collected in this study were all herb species, while the ones from all over the world included trees, shrubs and herbs. Generally, plants with different life forms have selectivity for the absorption of different nitrogen sources in soil, which leads to significant differences in δ^15^N among plants with different life forms, manifested as arbor > shrub > herb [4,32].

**Figure 2:**
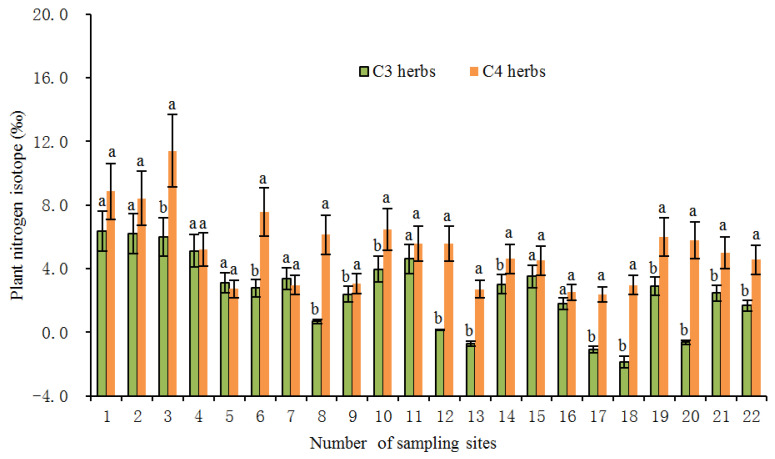
Average foliar δ^15^N of C3 and C4 herbs at each sampling site in the study transect. The numbers of the sampling sites correspond to the serial number in Table 3. All the values are represented as mean ± SD (standard deviation) at each sampling site. Different letters at each site indicate significant differences according to Duncan’s single-factor variance test at the 5% level.

**Figure 3:**
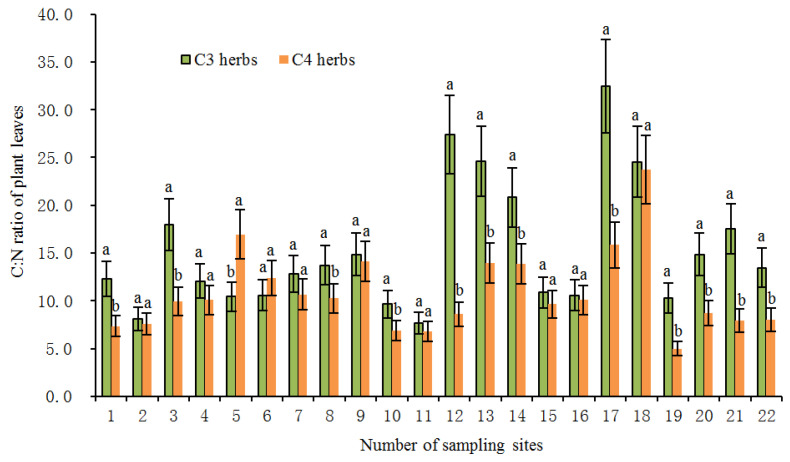
The C: N ratios of C3 and C4 herbs at each site in the study transect. The numbers of the sampling sites correspond to the serial number in Table 3. All the values are represented as mean ± SD (standard deviation) of each sampling site. Different letters at each site indicate significant differences according to Duncan’s single-factor variance test at the 5% level.

**Figure 4:**
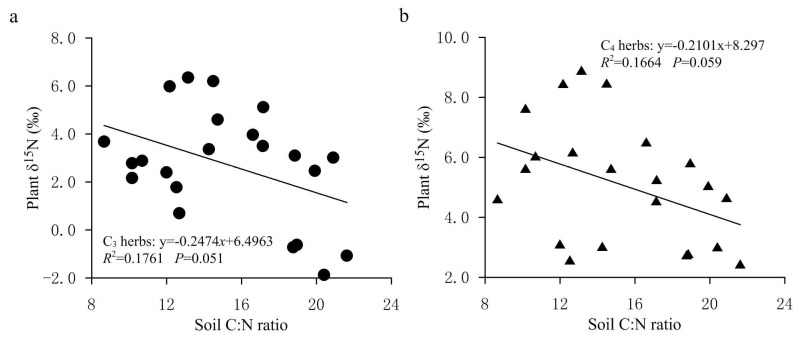
The changes in the δ^15^N values for C3 and C4 herbs with soil C: N ratio in the study area. (**a**) Relationship between δ^15^N values of C3 herbs and soil C: N ratio. (**b**) Relationship between δ^15^N values of C4 herbs and soil C: N ratio.

In the present study, the average foliar δ^15^N value of all investigated herbs was 4.06‰ (*n* = 231). Among them, the average value of δ^15^N of C3 herbs was 3.27‰ (*n* = 151), which was significantly lower (*p* < 0.01) than the average value of 5.55% of C4 herbs (*n* = 80). Within the same sampling location, the δ^15^N value of C4 herbs was significantly higher than that of C3 herbs at most sampling sites (Figure 2), illustrating a different N use by the two types of herbs with different photosynthetic pathways. The higher δ^15^N values for C4 herbs than for C3 herbs were consistent with previous studies [20,24,25,28]. For instance, the results from the secondary grassland in South China and the arid and semi-arid grasslands of North China showed that C3 plant δ^15^N values were significantly more depleted than C4 plant δ^15^N values [20,24]. The reasons why the δ^15^N value of C4 herbs was higher than that of C3 herbs could be explained from the following two aspects: On one hand, foliar δ^15^N is usually affected by the difference in carbon and nitrogen metabolism between different photosynthetic plants. Due to the differences in the photosynthetic nitrogen utilization rate between C3 and C4 plants, there are also differences in N-use efficiency. In general, C3 plants have more advantages in using nitrogen than C4 plants under drier conditions [26,33]. Plants with lower N-use efficiency have higher N concentrations and higher δ^15^N values [27,34]. As shown in Figure 3, the C:N ratios of C4 herbs were significantly lower than those of C3 herbs at most sampling sites, which indicated that C4 herbs have lower N utilization efficiency and higher foliar N concentrations than C3 herbs under similar N supply conditions. This was confirmed by findings of previous studies that soil organic matter derived from C4 grasses has a faster decomposition rate (attributable to lower C:N ratios) than those from C3 grasses [35]. Hence, within the same sampling site, it is not unexpected that C4 herbs are likely to show higher δ^15^N values than C3 herbs due to the lower leaf C:N ratios of C4 herbs. Additionally, there were significant differences in leaf δ^15^N among the three C4 species. The most conspicuous difference was that the *Amaranthus retroflexus* had substantially higher δ^15^N (8.31 ± 2.47‰, expressed by mean ± standard deviation, the same below) than the other two species (*Salsola collina*: 5.31 ± 2.72‰; *Setaria viridis*: 4.49 ± 2.92‰). Similarly, differences in nitrogen metabolism may also contribute to the differences we found in δ^15^N between the C4 species. We found that there were clear differences in leaf nitrogen content between them (*Amaranthus retroflexus*: 39.27 ± 7.64%; *Salsola collina*: 33.93 ± 6.74%; *Setaria viridis*: 25.30 ± 7.55%), implying that there is an obvious difference in nitrogen metabolism among the three C4 species. On the other hand, the δ^15^N values of individual plants are also determined to some extent by the isotopic ratio of the external source. Different photosynthetic plants usually have a preference for the uptake of available nitrogen sources (e.g., ammonium nitrogen and nitrate nitrogen) from soils, and the isotopic compositions of various nitrogen sources in the soils are obviously different as a result of local environmental conditions [36]. When there is more inorganic nitrogen, especially ammonium nitrogen, held by microorganisms in the soil, the availability of nitrogen sources (e.g., ammonium nitrogen) preferentially absorbed by plants is reduced. At this time, C3 plants will change their selection from ammonium nitrogen as the main nitrogen source to nitrate nitrogen as the main nitrogen source. The isotopic analysis of Aranibar et al. indicated that the δ^15^N of nitrate in soils was lower than that of ammonium at the same sites [4]. If C3 and C4 herbs prefer nitrate and ammonium, respectively, then the δ^15^N of C3 herbs would be lower than those of C4 herbs, as it was observed in most of the sampling sites (Figure 2). It has been reported that in some European grasslands, plants that preferred nitrate relative to ammonium had lower foliar δ^15^N than ones that preferred ammonium under controlled conditions [2]. However, the opposite result also appeared, namely plant species that preferred nitrate were more enriched in ^15^N [37]. One possible explanation is that nitrate in the soil may be more enriched than ammonium owing to the loss of gaseous N after nitrification. Moreover, changes in soil water may alter δ^15^N values in C3 and C4 plants by affecting their rooting depth and N availability and, thereby, the ^15^N signature of plant N sources, because nitrate and ammonium sources at different soil depths can vary in δ^15^N signature [8]. Other reports have indicated that the relative abundance of plant species in ecosystems may affect the composition and distribution of labile and recalcitrant N pools by changing the amount and quality of litter inputs, thereby changing soil nitrogen sources and affecting δ^15^N of C3 and C4 plants [35]. Unfortunately, we have not measured the ecological data in this regard, which limits our ability to determine the potential mechanism of nitrogen isotope differences between C3 and C4 photosynthetic pathways. In addition, we found that the δ^15^N of herbs decreased with the increase in soil C: N ratio along the transect, and the δ^15^N values of C3 herbs decreased more than those of C4 herbs under similar environmental conditions (Figure 4). This means that in the APZNC, nitrogen isotopes of different photosynthetic herbs have different responses to changes in soil C: N ratio. There are reports that if the soil C:N ratio is too high, the microbial decomposition and mineralization is slow, and the available nitrogen in the soil is consumed more, thus reducing the soil available nitrogen that can be absorbed by plants, and causing low soil ^15^N enrichment [38,39]. However, the response mechanism of δ^15^N for C3 and C4 herbs to the soil C: N ratio is still unclear. Since the δ^15^N values of nitrate nitrogen (${\mathrm{NO}}_{3}^{-}$) and ammonium nitrogen (${\mathrm{NH}}_{4}^{+}$) in the soils were not determined in this study, it limited further explanation on the causes of variation in plant δ^15^N. Therefore, it is necessary to study the effect of different nitrogen sources on plant δ^15^N in the future.

It is worth noting that the results of this study are contrary to those obtained from South Africa, the Mediterranean region and the Tengger Desert of China, that is, C3 plants had significantly higher δ^15^N values than C4 plants in the above areas [4,23,24]. For instance, in the Tengger Desert of China, Zhao et al. reported that δ^15^N was higher in C3 plants (varying from −4.45 to 3.66‰) than in C4 plants (varying from −7.56 to 1.08) [24]. However, we think it is difficult to compare the results of the two, mainly for two reasons. Firstly, all samplings in our study were conducted along a certain temperature gradient under the condition of similar precipitation (e.g., 400 mm isoline of MAP); whereas the sampling sites selected in South Africa, the Mediterranean region and the Tengger Desert of China were set along a certain precipitation gradient [4,23,24]. Therefore, the climatic conditions of the two were quite different. Secondly, in previous studies, except for the fact that the C4 plants analyzed were herbs, plants with the C3 pathway of photosynthesis were predominantly trees and shrubs, which was obviously different from C3 herbs investigated in this study. In order to adapt to the changes in environmental conditions, plants with different life forms usually show great differences in morphology and physiological traits, which further affect the foliar δ^15^N of plants. For example, the herbs in humid, semi-arid and mesic sites had lower δ^15^N values than those of trees from the same locations [40]. Therefore, from previous studies, it is difficult to distinguish the direct effects of the photosynthetic pathway and growth form on plant δ^15^N.

### 2.2. Responses of Foliar δ^15^N of C3 and C4 Herbs to Climatic Factors

#### 2.2.1. Response of Foliar δ^15^N Values of Herbs to MAP

**Figure 5:**
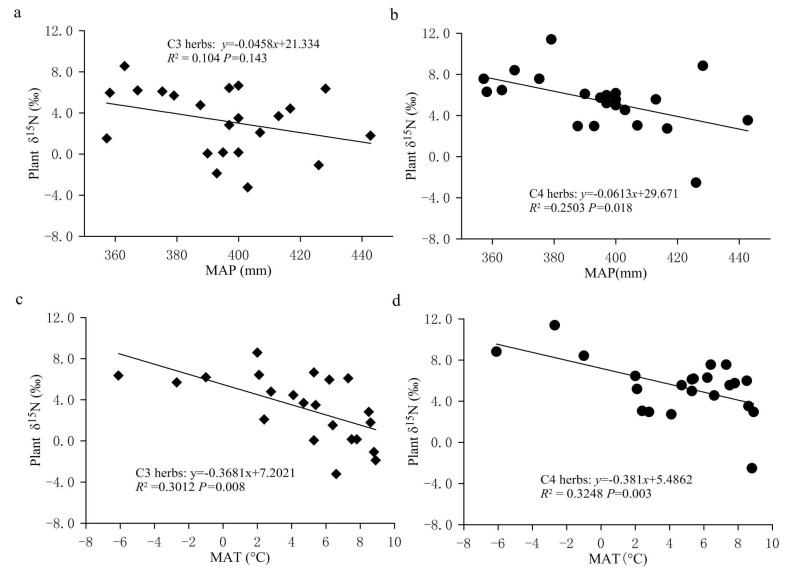
The changes in the δ^15^N values for C3 and C4 herbs with precipitation and temperature in the study area. (**a**) Relationship between δ^15^N values of C3 herbs and MAP. (**b**) Relationship between δ^15^N values of C4 herbs and MAP. (**c**) Relationship between δ^15^N values of C3 herbs and MAT. (**d**) Relationship between δ^15^N values of C4 herbs and MAT. All the values are expressed as the average of each sampling site. The same below.

**Figure 6:**
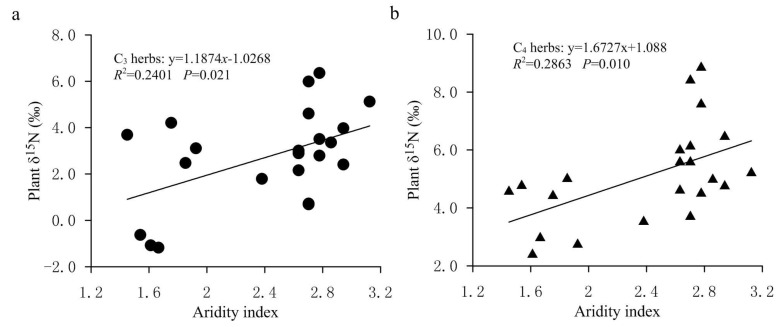
The changes in the δ^15^N values for C3 and C4 herbs with aridity index in the study transect. (**a**) Relationship between δ^15^N values of C3 herbs and aridity index. (**b**) Relationship between δ^15^N values of C4 herbs and aridity index.

In the FPENC, there was a negative correlation between foliar δ^15^N and MAP in both C3 and C4 herbs (Figure 5a,b). That is, foliar δ^15^N values of C3 and C4 herbs showed a linearly decreasing trend with increasing precipitation (significant for C4 herbs but not significant for C3 herbs). However, the δ^15^N responses of C3 and C4 herbaceous plants to precipitation changes were significantly different within the study area. Regression analysis showed that the slope of the relationship between δ^15^N and MAP was steeper in C4 herbs compared with C3 herbs. Over the entire range of MAP, for every 100 mm increase in MAP, the δ^15^N value of C3 herbs declined by approximately 4.58% while that of C4 herbs decreased by about 6.13%, indicating that the δ^15^N values of C3 herbs were less responsive to drought than those of C4 herbs. Figure 6 showed that, on the study transect, the change rate of foliar δ^15^N of C4 herbs with AI was significantly greater than that of C3 herbs. This can also be confirmed by the results of partial correlation analysis between δ^15^N values of herbs and climate factors. Table 1 shows that the partial correlation between the δ^15^N value of C4 herbs and the MAP in our study is significantly better than that between the δ^15^N value of C3 herbs and the MAP. The reason may be that the two plant types have a different N metabolism because the differences in carboxylation reactions lead to disparate isotopic fractionation between the two photosynthetic pathways (C3 and C4 plants). In addition, due to the different photosynthetic pathways’ environmental controls, the N absorption by C4 plants might be more affected by the competitive pressure of neighboring plants and soil microorganisms than C3 plants along the transect, resulting in the variation in C4 plants’ δ^15^N being more sensitive to precipitation [26]. This demonstrates that the responses of plant δ^15^N to environmental changes may also be dependent on the photosynthetic pathways [41]. Although the δ^15^N value of C3 and C4 herbs was correlated with the MAP, the simple relationship between δ^15^N signal and MAP only explained a small amount of the observed variance in the transect. The MAP accounted for just 10.40% and 25.03% of the nitrogen isotope variations in C3 and C4 herbs, respectively (Figure 5a,b). This means that δ^15^N signature of herbs has a limited indicative significance for the variation in precipitation across the study region. Multiple regression analysis (Table 1) also shows that the regression coefficients of the MAP against plant δ^15^N values are only −0.035 (C3 herbs) and −0.053 (C4 herbs), respectively, which again indicates that the influence of precipitation on the δ^15^N of herbs is limited in the study transect. This phenomenon may be related to the fact that there is no obvious precipitation gradient on the study transect. This is because although precipitation is considered one of the key factors to determine plant δ^15^N in arid and semi-arid environments, most of the plant samples in this study were collected along the 400 mm isoline of MAP, and the MAP between the sampling sites varies from 345.0 to 442.8 mm, with an average value of 394.77 ± 24.07 mm. Therefore, there is no significant difference in the effect of precipitation on foliar δ^15^N of herbs due to little difference in precipitation among various sampling sites on the study transect.

However, contrary to our findings, Swap et al. and Zhao et al. reported that the relationship between plant δ^15^N and precipitation in Southern Africa and in the Tengger Desert of China was much stronger and steeper in C3 than C4 plant [23,24]. The reason for this phenomenon may be that the C3 plant samples used included a large number of woody plants, so it was difficult to distinguish the effects of the photosynthetic pathway on plant δ^15^N values. As for the reason why δ^15^N in C3 grass was more negative in response to increased precipitation than C4 grass in Australian grasslands, Murphy and Bowman did not give a reasonable explanation [41].

This negative relationship between herb δ^15^N and precipitation in this study area is consistent with previous research on local (e.g., Northeast China Transect (NECT), Southern Africa, and Loess Plateau in Northwest China), regional and global scales [4,10,11,14,21,23,26,30,41]. For example, a study conducted across a 1200 km transect of Inner Mongolian grasslands showed that the δ^15^N of two grass species was negatively correlated with MAP [42]. The study by Craine et al. based on over 11000 non-N2-fixing plants worldwide is another example of the pattern of increasing δ^15^N with decreasing MAP [10]. However, the response degree of plant δ^15^N to precipitation varies with different study areas. In the study area, the response of plant δ^15^N to the MAP is significantly greater than that obtained in the Loess Plateau of China (plant roots: −1.1%/100 mm; plant residue: −1.4%/100 mm), NECT (C3 herbs: −1.3%/100 mm; C4 herbs: −1.1%/100 mm) and South Africa (−0.47/100 mm) [4,30].

**Figure 7:**
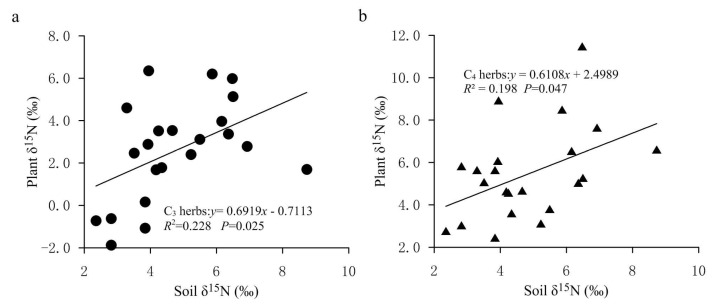
Correlations of δ^15^N of C3 and C4 herbs with surface soil δ^15^N in the study area. (**a**) Relationship between δ^15^N values of C3 herbs and soil δ^15^N values. (**b**) Relationship between δ^15^N values of C4 herbs and soil δ^15^N values.

At present, there are many explanations for the decrease in plant δ^15^N with increasing precipitation. Sutton et al. explained the decrease in plant δ^15^N caused by the increase of precipitation by describing that plants directly absorbed ${\mathrm{NH}}_{4}^{+}$ in atmospheric precipitation through leaf stomata or epidermis, and the δ^15^N from ammonium nitrogen in precipitation is often more negative than that from nitrate nitrogen, which leads to the reduction in plant δ^15^N [43]. A popular explanation for higher plant δ^15^N in lower-precipitation areas is mainly related to greater N losses at drier sites and to ^15^N enrichment in soil because of ^15^N-depleted N loss through volatilization, denitrification and leaching [18]. In other words, dry sites have a more open N cycle with a greater importance of inputs and outputs compared to humid sites. This is because the acquisition of nitrogen by plants mainly comes from the absorption of soil inorganic nitrogen (ammonium nitrogen and nitrate nitrogen), while the impact of increased precipitation on soil available nitrogen is mainly realized by reducing microbial activity and changing the relative content of ammonium nitrogen and nitrate nitrogen in the soil. Therefore, the nitrogen isotope fractionation occurs during the above process. In general, with the decrease in the aridity index, the utilization efficiency of ${\mathrm{NO}}_{3}^{-}$ in soil increases, and more ${\mathrm{NO}}_{3}^{-}$ is absorbed by plants and stored in soil nitrogen pool, resulting in an increase in ${\mathrm{NO}}_{3}^{-}$ ions and ^15^N-depleted nitrogen in soil. Meanwhile, with the increase in soil moisture, soil microbial activity is reduced and the soil nitrification process is inhibited, which causes the availability of soil inorganic nitrogen to decrease and inhibits ^15^N enrichment in the soil nitrogen pool, thus resulting in negative δ^15^N values in soils. In the relatively arid area of the FPENC, soil microbial activity increases due to the reduction in annual precipitation and high rates of evaporation, which leads to enhanced ammonification and openness of the nitrogen cycle in the soil. Thus, the ammonium nitrogen in the soil is easy to volatilize on the soil surface, thereby enriching ^15^N in the soil nitrogen pool. Because plant δ^15^N was positively correlated with soil δ^15^N in our study (Figure 7), the δ^15^N values of herbs showed a negative correlation with increasing precipitation, but this was weak for C3 herbaceous plants (Figure 5a,b). These paralleled δ^15^N values between plants and soil support the fact that soil N was the dominant N source for the C3 and C4 herbs in the present study, and also indicate an effective internal recycling of nitrogen within the plant–soil system. 

**Figure 8:**
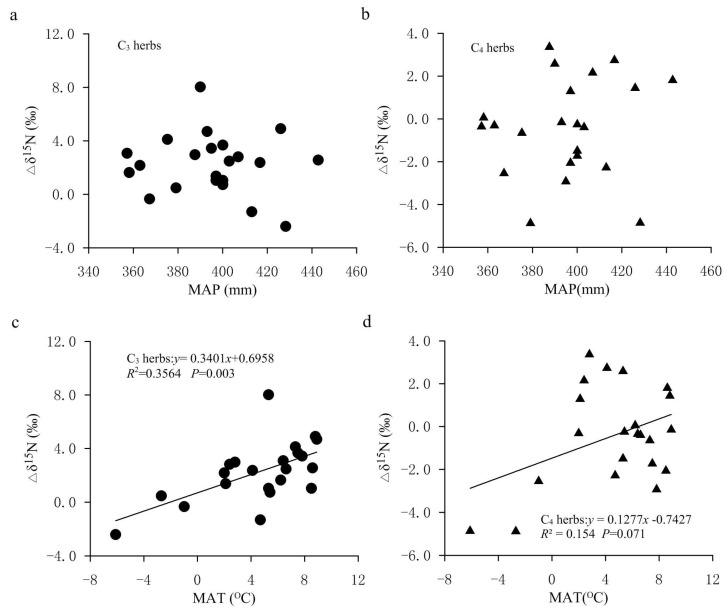
The isotopic ^15^N (Δδ^15^N) differences between soil and plants along the climatic gradient in the study area. (**a**) Relationship between Δδ^15^N (δ^15^N difference between soil and C3 herbs) and MAP. (**b**) Relationship between Δδ^15^N (δ^15^N difference between soil and C4 herbs) and MAP. (**c**) Relationship between Δδ^15^N (δ^15^N difference between soil and C3 herbs) and MAT. (**d**) Relationship between Δδ^15^N (δ^15^N difference between soil and C4 herbs) and MAT.

The response of nitrogen isotope discrimination to climate factors is more complex compared with carbon isotope discrimination. Precipitation and nitrogen availability usually play an important role in the nitrogen isotopic discrimination in soil and plants [44]. In our study, the negative effect of MAP on plant δ^15^N values may be due to the reduction in net nitrogen isotope discriminations (∆δ^15^N) in soil. We found that in the FPENC, the ∆δ^15^N values (the δ^15^N difference between soil and plants) decreased with the increase in MAP, but not significantly (Figure 8a,b). This was consistent with the patterns obtained by predecessors in the dryland ecosystem (MAP < 500 mm), but contrary to the patterns obtained in areas with MAP > 800 mm [12,20,44]. This is because a higher MAP is conducive to the loss of nitrogen, which may lead to higher δ^15^N values in wetter soil, but the increased leaching with the increase in MAP can also discriminate against ^15^N [18,23].

#### 2.2.2. Response of Foliar δ^15^N Values of Herbs to MAT

Temperature is another important environmental factor affecting nitrogen isotope fractionation in plants. Lots of studies have indicated that at regional and global scales, foliar δ^15^N of terrestrial plants tended to increase with increasing temperature. For example, Craine et al. synthesized foliar δ^15^N from global sites and found that foliar δ^15^N increased with increasing MAT [2]. Martinelli et al. reported that the average δ^15^N of plants from tropical regions was 6.5% higher than that from temperate regions (3.7 vs. −2.8%) [16]. In addition, increased plant δ^15^N with rising temperature was also observed along the East African Rift Zone in Ethiopia [15], the Dongling Mountain in Beijing [45], Gongga Mountain [46] and over East China [22]. In this study, however, the variation in foliar δ^15^N of herbs showed a significant negative trend with increasing MAT (Figure 5c,d and Table 1). The regression analysis of δ^15^N values of C3 and C4 herbs and MAT led to a correlation that was extremely significant (*p* < 0.001), but the temperature only explained 30.12% (C3 herbs) and 32.48% (C4 herbs) of the observed variation (Figure 5c,d), demonstrating that there was no significant difference in the responses of δ^15^N value of C3 and C4 herbs to temperature. When the MAT increased by 1 °C, the foliar δ^15^N values of C3 and C4 herbs decreased by 0.368% and 0.381%, respectively. In the present study, the variation trend of δ^15^N values of herbs with temperature was opposite to that for all plants at a global scale [10,14], but similar to the results that foliar δ^15^N was negatively correlated with increasing MAT in the Loess Plateau of China [21], the grasslands of the NECT and a climatic gradient transect in South China [20,30,47]. For instance, Feng et al. found that the δ^15^N values of both the C3 and C4 plants declined significantly with increasing MAT. There are three explanations for the positive correlation between plant δ^15^N and temperature. One explanation is that hot environments have a greater proportion of N being lost through fractionating pathways and a more open soil N cycle. More specifically, with the increase in temperature, an increasing fraction of soil N losses are ^15^N-depleted forms [4]; the other explanation for this positive relationship may be related to net nitrification rates. The activity of nitrifying bacteria is usually very sensitive to temperature and increases with rising temperature. Thus, in warmer locations, more ${\mathrm{NH}}_{4}^{+}$ that is more easily absorbed by plants is converted into ${\mathrm{NO}}_{3}^{-}$ which is relatively more difficult to be utilized by plants, resulting in plant δ^15^N values closer to zero [22]. Meanwhile, the net mineralization caused by microorganisms in the soil is enhanced with the increase in temperature. The synergistic effects of the above two effects may make plant δ^15^N and temperature positively correlated. The third explanation is due to the correlation observed at local scale between nitrogen availability and foliar δ^15^N; this positive global relationship between temperature and plant δ^15^N is interpreted as the increased availability of nitrogen in warm environments. Because sites with higher nitrogen availability are more likely to have plants with higher nitrogen concentrations, plant nitrogen concentrations tend to correlate positively with foliar δ^15^N [10]. As for the negative correlation between plant δ^15^N and increasing temperature in the Loess Plateau of China and the NECT, some researchers attributed it to the fact that the significant negative effect of increasing precipitation on plant δ^15^N in the same period of “rain and heat” exceeded the positive effect of rising temperature on plant δ^15^N [21,30]. This means that the negative correlation trend with temperature cannot truly reflect the relationship between plant δ^15^N and temperature in the Loess Plateau and the NECT.

In the present study, we believed that temperature was an important factor affecting the variation in plant δ^15^N, and it was possible that plant δ^15^N values decreased with the increase in temperature. There may be three reasons for this. First, the surveyed transect spans a wide range (the north–south distance is about 1900 km, and the east–west distance about 1500 km) where the MAT was −6.1 to 8.9 °C (Table 3). The difference between the maximum and minimum temperatures was about 15.0 °C, and there was an obvious temperature gradient from low to high along the northeast to southwest transect. However, the difference in MAP among the various sampling sites on this transect is small, which can basically be regarded as the same (Table 3). Second, by comparing the relationships between temperature/precipitation and longitude/latitude in the transect, it can be observed that both the MAT and the MAP showed a decreasing trend with the increase in latitude and longitude. Among them, the MAT and longitude/latitude were highly correlated. The MAT decreased by 0.43 °C and 0.74 °C for every 1 degree increase in longitude and latitude, respectively, whereas the MAP and longitude/latitude did not show a significant correlation (Table 2). This implies that the influence of temperature with longitude and latitude on plant δ^15^N should be greater than that of precipitation. The multiple linear regression analysis on foliar δ^15^N of herbs and climate factors showed that the absolute value of the regression coefficient of the MAT was much larger than that of the MAP (Table 1), indicating that on this transect, temperature is the key factor affecting plant δ^15^N values. This means that the change in nitrogen isotopes of plants can indicate information regarding temperature change in this region to a certain extent. Thirdly, previous studies also concluded that with the increase in temperature, plant δ^15^N decreased or did not change significantly [41,48]. For instance, on the global scale, foliar δ^15^N decreases with increasing MAT when the temperature is below −0.5 °C [49]. Yi and Yang found that the δ^15^N of alpine meadow plants gradually increased with the decrease in temperature, but it was not significant [50]. Our previous study in the Dongling Mountain of Beijing also showed that when the temperature was less than 3.5 °C, the δ^15^N values of herbs decreased with the increase in temperature, and while the temperature was above the value, the δ^15^N increased with the increase in temperature [45]. As for the increase in foliar δ^15^N with decreasing temperature, it may be that lower temperatures increased the viscosity of soil solution or water in plants and caused soil or plants to be under water stress (i.e., physiological drought), thus increasing plant δ^15^N values. It is worth mentioning that in this study, although the significant negative effect of temperature on leaf δ^15^N occurred, the net N isotope discrimination (Δδ^15^N) in plants and soil increased with the increase in MAT (Figure 8c,d). This showed that soil ^15^N-depleted gaseous N losses were greater relative to N input from plant fixation in our study, which also means that N isotope discrimination in plants and soil to temperature variable is very complex.

## 3. Methods

### 3.1. Sample Collection, Processing and Nitrogen Isotope Determination

**Figure 9:**
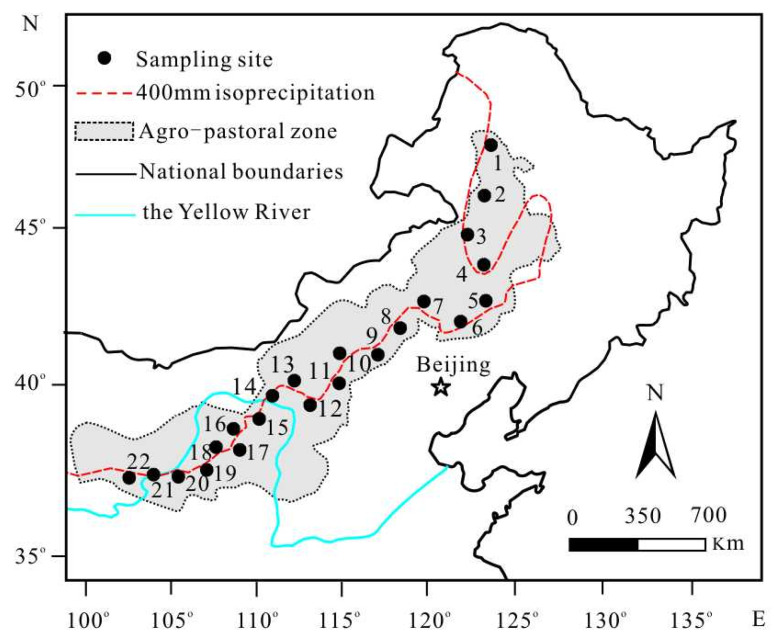
The locations of the sampling sites in the study area. The Arabic numerals in Figure 9 are the numbers of sampling sites, which correspond to the serial numbers in Table 3.

From July to August 2020, we established a northeast–southwest transect across the 400 mm isoline of MAP in the APZNC (Figure 9). The transect is approximately 1500 km long and covers approximately 11° latitude and 18° longitude (36.92–48.20° N and 104.20–122.03° E). Along the entire transect, a total of 22 representative sampling sites were selected 500–1000 m away from major roads and human settlements, without grazing and other anthropogenic disturbances (Figure 9). The longitude, latitude and altitude of each sampling location were measured with GPS (eTrex Venture, Garmin, Kansas City, MO, USA). More details of all sampling sites used in this study are presented in Table 3. At each site, three quadrats with an area of 2.0 m × 2.0 m were randomly selected. Within each quadrat, five to seven mature and healthy individuals of each dominant herb species or eurytopic species were collected, and then combined into one sample of the same species. After air drying in the field, collected plant samples in each site were carefully classified according to their photosynthetic pathway as both C3 and C4, and then stored in envelopes separately. While collecting plant samples, the surface soils (0–10 cm depth) of the corresponding quadrat were also collected systematically by a soil gauge (2.5 cm diameter) in three repetitions. After removing weeds, fine roots, gravel and other sundries, three drill soils were mixed to form one soil sample in each quadrat and put into plastic self-sealing bags. A total of 241 herbaceous plant samples (including 161 C3 plant samples and 80 C4 plant samples) and 66 soil samples were collected in this study. 

The plant samples were brought back to the laboratory, dried at 65 °C for 48 h to achieve a constant weight, and then ground to pass through an 80 mesh sieve. Air-dried soil samples were ground to sieve through a 2 mm mesh, and then soaked with 0.5 mol/L hydrochloric acid solution for 24 h to remove carbonates in the soil, and ultimately, the filtered soil extract was used to measure soil δ^15^N. For the prepared plant and soil samples, the δ^15^N, C and N concentrations were determined using a Finnigan MAT-Delte^plus^XP mass spectrometer (Thermo Finnigan, San Jose, CA, USA) with an automatic continuous-flow Flash EA1112 elemental analyzer (Thermo Finnigan, San Jose, CA, USA), and then the mean value per sampling site was calculated. The nitrogen isotopic composition is defined in per mil (%) relative to atmospheric N_2_ and expressed as:

(1) $$\delta^{15}N\;\left(‰\right)=\left[\left(\frac{R_{\mathrm{sample}}}{R_{standard}}\right)-1\right]\times 1000$$

where $R_{\mathrm{sample}}$ and $R_{standard}$ represent the ^15^N/^14^N ratios of the sample and standard, respectively. The measurement accuracy of nitrogen isotopes was ± 0.3%.

### 3.2. Meteorological Data of Sample Sites

Two main climatic variables (MAT and MAP) were used to explore the climatic controls on response patterns of δ^15^N in C3 and C4 herbs. The MAT and MAP were obtained from local weather stations of the China Meteorological Data Service Centre and Chinese Natural Resources Database. Simultaneously, the aridity index (AI) of each sampling site was calculated by Equation (2).

(2) $$\mathrm{AI}=\frac{{\mathrm{PT}}_{0}}{R_{0}}=\left(58.93\times \frac{1}{12}\sum_{1}^{12}T\right)/R_{0}$$

where ${\mathrm{PT}}_{0}$ is the potential evapotranspiration (mm),${\;R}_{0}$ is the annual precipitation (mm) and T is the monthly average temperature (If the monthly average temperature exceeds 30 °C, it shall be calculated as 30 °C; when the monthly average temperature is lower than zero °C, it is calculated as 0 °C). The above climatic data were the average of observed data during the 30 years from 1990 to 2020. 

### 3.3. Statistical Analysis

Ordinary least squares regression (OLSR) was used to examine the responses of plant δ^15^N to climatic factors as well as to soil δ^15^N. In the process of analysis, only non-N_2_-fixing herbaceous species were selected for this study. Meanwhile, ten samples of five annual C3 herbs were excluded in order to eliminate the influence of leaf age on plant δ^15^N values. In other words, the C3 plant samples involved in the analysis were all biennial or perennial herbs. Considering the correlation between precipitation and temperature in the study area, partial correlation and multiple linear regression analyses based on the Akaike information criterion (AIC) were used to explore the relationships of plant δ^15^N against climatic variables such as temperature and precipitation, so as to distinguish the effects of temperature and precipitation on plant δ^15^N. In addition, one-way ANOVA (analysis of variance) was performed to test whether there was a significant difference in the average δ^15^N values between C3 and C4 herbs. All statistical analyses were conducted with SPSS 13.0 software (SPSS Inc., Chicago, IL, USA).

## 4. Conclusions

Through the investigation of the foliar δ^15^N of herbs in the APZNC and the responses of plant δ^15^N to the MAP and MAT, the following conclusions were preliminarily drawn: (1) The C3 and C4 herbs in the APZNC exhibited an obvious difference in their δ^15^N signatures. The δ^15^N values of all herbs investigated varied from −5.5% to 15.25%. Among them, the δ^15^N value range of C3 herbs (−5.5~15.00%) was wider than that of C4 herbs (−2.17~15.25%), but its average value (3.27%) was significantly lower than that of C4 herbs (5.55%). (2) From northeast to southwest along the transect, the δ^15^N of herbs decreased linearly and significantly with the increase in precipitation, which is consistent with previous related studies. However, the ability of MAP to explain the nitrogen isotope changes in C3 and C4 herbs was weak, only 10.40% (C3 herbs) and 25.03% (C4 herbs), respectively, indicating that the influence of precipitation on plant δ^15^N is limited in this transect. (3) In the transect, the δ^15^N of both C3 and C4 herbs decreased significantly with the increase in MAT. The δ^15^N of C3 and C4 herbs decreased by 0.368‰ and 0.381‰, respectively, for every 1°C increase in MAT. There was no significant difference in the δ^15^N responses of C3 and C4 herbs to temperature. However, the interpretation ability of temperature to the change in δ^15^N of C3 herbs was significantly higher than that of precipitation, which suggests that temperature is a key factor affecting foliar δ^15^N of herbs in this transect. The above findings increased our knowledge of the δ^15^N signatures of C3 and C4 herbs and their responses to climate change, which could facilitate global change researchers to use plant δ^15^N as a proxy to explain the process of the nitrogen cycle and gain an insight into climate dynamics of the past. Since the sampling area was selected along the 400 mm isoline, the range of tested precipitation is very narrow. In addition, the δ^15^N values of nitrate nitrogen and ammonium nitrogen in the soils were not measured in this study. Therefore, future work is needed across a larger scale with a wide range of precipitation. Moreover, it is necessary to study the effects of different nitrogen sources on plant δ^15^N.

## Figures and Tables

**Table 1:** Partial correlation and multiple regression analysis of foliar δ^15^N of herbs and climate parameters.

Variable Name	MAT(*x*_1_)		MAP(*x*_2_)		Multiple Regression Equation
	SCC	PCC	SCC	PCC	
δ^15^N of C3 herbs (*y*)	−0.368 **	−0.365 **	−0.188 *	−0.183 *	y = −0.386*x*_1_ − 0.035*x*_2_ + 18.98 ***
δ^15^N of C4 herbs (*y*)	−0.381 **	−0.337 **	−0.361 **	−0.313 **	y = −0.310*x*_1_ − 0.053*x*_2_ + 27.91 ***

Note: * means significant at the level of *p* < 0.05, ** means significant at the level of *p* < 0.01, *** means significant at the level of *p* < 0.001. SCC: Simple correlation coefficient; PCC: Partial correlation coefficient.

**Table 2:** Regression analysis of climate parameters and geographic variables.

Climate Parameters	Longitude (°)		Latitude (°)	
	Regression Equation	Correlation Coefficient	Regression Equation	Correlation Coefficient
MAT(°C)	*y* = −0.43*x* + 54.1	−0.62 **	*y* = −0.74*x* + 35.3	−0.81 **
MAP(mm)	*y* = −0.25*x* + 425	−0.06^NS^	*y* = −0.31*x* + 410	−0.06 ^NS^

Note: ** indicates significant at *p* < 0.01 level; NS means not significant; *y* is a climate variable, representing the MAT or MAP; *x* is a geographical variable, representing longitude or latitude.

**Table 3:** The information of the sampling sites and plant species collected at each site.

No.	Site	Longitude (°)	Latitude (°)	MAT (°C)	MAP (mm)	Sample Size (n)	Grassland Type	Species Names
1	Jinhe	121.29	48.20	−6.1	428	9 (3)	Meadow grassland	1, 2, 3, 4, 5, 6, 7, 8
2	Hailar	119.14	47.23	−1.0	367	6 (4)	Meadow grassland	3, 6, 7, 8, 9, 10, 11, 12
3	Arshan	119.93	47.14	−2.7	392	8 (4)	Typical grassland	3, 4, 5, 6, 7, 8, 10, 13, 14
4	HRFB	121.58	46.05	2.1	397	3 (5)	Meadow grassland	2, 3, 6, 7, 8
5	Ulanhot	122.03	46.04	4.1	416	6 (6)	Meadow grassland	3, 4, 5, 6, 7, 8, 13, 14, 15
6	BYHS	121.27	45.04	7.3	357	8 (5)	Typical grassland	1, 3, 6, 8, 9, 14, 16, 17, 18
7	Jarud Banner	120.90	44.57	2.8	387	9 (4)	Meadow grassland	2, 3, 5, 7, 8, 12, 13, 18, 19
8	Bairin Zuoqi	119.60	43.98	5.3	390	11 (4)	Typical grassland	3, 4, 5, 6, 7, 8, 9, 12, 13, 14, 18
9	Duolun	116.47	42.18	2.4	407	14 (4)	Meadow grassland	1, 3, 5, 6, 7, 8, 9, 13, 18
10	Bai Banner	115.12	42.24	2.0	363	4 (4)	Typical grassland	3, 5, 6, 7, 8, 13, 14
11	Fengzhen	113.45	40.26	4.7	413	12 (3)	Meadow grassland	1, 3, 4, 5, 6, 7, 8, 9, 12, 13
12	Jungar Banner	110.26	39.35	7.5	400	4 (1)	Meadow grassland	3, 6, 8, 13, 16
13	Ordos	110.47	39.03	6.4	345	3 (1)	Typical grassland	2, 6, 3, 7
14	Horo Banner	110.05	39.17	6.2	365	3 (1)	Typical grassland	3, 5, 6, 7
15	Dongsheng	109.98	39.38	5.4	400	6 (4)	Typical grassland	3, 4, 5, 6, 8, 10, 14
16	Youyu	112.27	39.03	8.6	443	8 (5)	Meadow grassland	1, 2, 3, 5, 6, 7, 8, 13, 14
17	Hequ	111.15	39.02	8.8	426	6 (2)	Meadow grassland	3, 6, 9, 12, 8, 13, 14, 15,
18	Shenmu	109.54	38.24	8.9	393	3 (3)	Meadow grassland	3, 4, 6, 7, 8, 13, 14
19	Hengshan	109.17	37.36	8.5	398	9 (4)	Typical grassland	1, 3, 4, 6, 7, 8, 9, 11, 14
20	Jingbian	108.50	37.28	7.8	395	6 (4)	Typical grassland	1, 2, 3, 6, 7, 8, 13, 14
21	Xiji	105.44	37.57	5.3	400	8 (5)	Meadow grassland	2, 3, 5, 6, 8, 14, 18
22	Yuzhong	104.20	36.92	6.6	403	5 (4)	Meadow grassland	3, 5, 6, 7, 8, 14, 18

Note: MAT is mean annual temperature and MAP is mean annual precipitation. The numbers outside and inside the brackets are the sample sizes of C3 herbs and C4 herbs, respectively. HRFB: Horqin Right Front Banner; BYHS: Bai Yin Hu Shuo. The Arabic numbers in the column of “species names” represent the plant species names. The plant species are as follows.1: *Spiraea salicifolia L*.; 2: *Festuca arundinacea Schreb*.; 3: *Leymus chinensis* (*Trin*.) *Tzvel*.; 4: *Stipa grandis P.A. Smirn*.; 5: *Artemisia lavandulaefolia DC*; 6: *Plantago depressa Willd*.; 7: *Salsola collina Pall*.; 8: *Setaria viridis* (L.) *Beauv*.; 9: *Elymus dahuricus Turcz*.; 10: *Stipa bungeana Trin*.; 11: *Bothriochloa ischaemum* (L.) *Keng*; 12: *Artemisia frigida Willd*.; 13: *Artemisia capillaris Thunb*.; 14: *Amaranthus retroflexus* L.; 15: *Lepidium apetalum*; 16: *Melilotus suaveolens Ledeb*.; 17: *Lolium perenne* L.; 18: *Agropyron cristatum* (L.) *Gaertn*.; 19: *Equisetum ramosissimum*.

## Data Availability

The data will be provided by the corresponding author upon request.
